# Consistency and variability in functional localisers

**DOI:** 10.1016/j.neuroimage.2009.03.014

**Published:** 2009-07-15

**Authors:** Keith J. Duncan, Chotiga Pattamadilok, Iris Knierim, Joseph T. Devlin

**Affiliations:** a Cognitive, Perceptual and Brain Sciences and Institute of Cognitive Neuroscience, University College London, 26 Bedford Way, London, WC1H 0AP, UK; b Fonds de la Recherche Scientifique-FNRS & Université Libre de Bruxelles, Belgium; c Université Pierre et Marie Curie, and Ecole Normale Supérieure, Paris, France

**Keywords:** fMRI, Reading, Object recognition, Posterior fusiform gyrus, Functional localiser, Occipito-temporal cortex

## Abstract

A critical assumption underlying the use of functional localiser scans is that the voxels identified as the functional region-of-interest (fROI) are essentially the same as those activated by the main experimental manipulation. Intra-subject variability in the location of the fROI violates this assumption, reducing the sensitivity of the analysis and biasing the results. Here we investigated consistency and variability in fROIs in a set of 45 volunteers. They performed two functional localiser scans to identify word- and object-sensitive regions of ventral and lateral occipito-temporal cortex, respectively. In the main analyses, fROIs were defined as the category-selective voxels in each region and consistency was measured as the spatial overlap between scans. Consistency was greatest when minimally selective thresholds were used to define “active” voxels (*p* < 0.05 uncorrected), revealing that approximately 65% of the voxels were commonly activated by both scans. In contrast, highly selective thresholds (*p* < 10^− 4^ to 10^− 6^) yielded the lowest consistency values with less than 25% overlap of the voxels active in both scans. In other words, intra-subject variability was surprisingly high, with between one third and three quarters of the voxels in a given fROI not corresponding to those activated in the main task. This level of variability stands in striking contrast to the consistency seen in retinotopically-defined areas and has important implications for designing robust but efficient functional localiser scans.

*In choosing a localizer to define an ROI, the researcher is making an ontological assumption that this localizer contrast picks out a meaningful functional unit in the brain (i.e., a natural kind). Like other ontological assumptions in science, the utility of a particular functionally defined ROI is determined by the consistency of the data that emerge from it and the richness of the theoretical progress those data support.*Saxe et al. (2004), 91–92.

## Introduction

Increasingly, functional neuroimaging studies are moving away from traditional brain mapping studies designed to identify the cortical topography of a function (Ψ) and towards designs that investigate the response properties of specific neuroanatomical regions. This approach requires a robust method for identifying the region under investigation, however, macro-anatomic landmarks are not especially good predictors of functionally homogenous cortical fields (Amunts et al., 2000; Farrell et al., 2007; Uematsu et al., 1992). The early visual fields are a good example. V1 is primarily located in the calcarine sulcus but its borders do not correspond to clear sulcal landmarks while V2 and V3 are even more difficult to distinguish based purely on local landmarks (Amunts et al., 2000; Wohlschlager et al., 2005). The inability to define a region unambiguously is a major impediment to investigating it. Consequently, a typical solution is to localise the region functionally based on its response properties, for instance, using retinotopy (Larsson and Heeger, 2006; Sereno et al., 1995), somatotopy (Blankenburg et al., 2006; Huang and Sereno, 2007; Pulvermüller et al., 2006), or tonotopy (Bilecen et al., 1998; Talavage et al., 2004; Wessinger et al., 1997; Wessinger et al., 2001). Even higher order association areas can be defined in this way with “functional localisers” routinely used to identify the set of voxels sensitive to faces (Downing et al., 2006; Haxby et al., 2001; Jiang et al., 2007; Levy et al., 2001; O'Craven and Kanwisher, 2000; Yovel et al., 2008), speech (Miller and D'Esposito, 2005; Szycik et al., 2008), objects (Culham et al., 2003; Eger et al., 2008; Haxby et al., 2001; Jiang et al., 2007; Kourtzi and Kanwisher, 2000; Levy et al., 2001; Yovel et al., 2008), body parts (Downing et al., 2006; Saxe et al., 2006b), scenes (Downing et al., 2006; Epstein and Kanwisher, 1998), or written words (Baker et al., 2007; Ben-Shachar et al., 2007). In most cases this involves collecting additional scans in which participants perform a different task solely for the purpose of functionally identifying the anatomical region and then using it in summary mode as a way of evaluating the response profile of a functionally defined region-of-interest (fROI).

Although there is some debate regarding the most efficient method for doing this (Friston et al., 2006; Saxe et al., 2006a), relatively little attention is paid to the validity of a key underlying assumption — namely, how consistent are the localisations? Obviously the tacit assumption is that the same task in the same subject will identify essentially the same set of voxels despite various sources of physiological and scanner noise (Aguirre et al., 1998; Handwerker et al., 2004; Kruger and Glover, 2001). If there is considerable variability between runs within the same session, then the basic idea of functional localisation becomes suspect because the localised set of voxels may not correspond well to those being tested in the main experimental run, decreasing sensitivity and increasing both false positives and false negatives.

One of the few studies to investigate this issue examined the consistency of activation for faces in the fusiform and occipital face areas (FFA and OFA), scenes in the parahippocampal place area (PPA), and body parts in the extrastriate body area (EBA) (Peelen and Downing, 2005). They found all stimuli produced peak voxels that were consistent in both location and *t*-value across runs. They did not, however, report the consistency of the activation itself, which is important because most studies that use functional data to identify a region-of-interest define it based on the cluster of voxels within a given anatomical area activated by a particular contrast (Downing et al., 2006; Grill-Spector et al., 2004; Jiang et al., 2007; Kanwisher et al., 1999; O'Craven and Kanwisher, 2000; Spiridon et al., 2006; von Kriegstein et al., 2008; Yovel et al., 2008). One study which did investigate the consistency of activation for faces found that although the location of the peak voxel was stable, there was less than 40% overlap^1^ in the number of active voxels between localiser runs (Kung et al., 2007), suggesting that functionally defined ROIs may be more variable than commonly assumed.

Here we had the opportunity to evaluate consistency and variability associated with functionally localising reading- and object-sensitive areas of left occipito-temporal cortex (OTC). As part of an on-going series of TMS studies, we used fMRI to localise a region of the ventral OTC associated with visual word recognition (Price and Mechelli, 2005) and a lateral OTC region associated with visual object recognition (Grill-Spector et al., 1999; Malach et al., 1995) in a fairly large sample of volunteers (*n* = 45). To empirically evaluate the assumption that functional localisation of category-sensitive cortical regions is robust and consistent, we calculated three different measures of consistency between two functional localiser runs: (1) the distance between peak voxels in the two runs; (2) the amount of spatial overlap in activations and (3) the amount of overlap in contiguously activated voxels within a spherical ROI centred on the peak voxel. The results illustrate considerable within-subject variability in the localisation of the two fROIs and call into question the validity of a key assumption underlying typical functional localiser scans.

## Materials and methods

### Participants

45 (23 M, 22 F) healthy, monolingual English speakers participated in an fMRI study as part of a neuro-navigated TMS study (Duncan et al., in press). Their ages ranged from 19 to 38 (mean = 25), and all were right handed with normal or corrected-to-normal vision. None had a personal or family history of any neurological disease, and each gave informed consent after the experimental procedures were explained. This experiment was approved by the Berkshire NHS Research Ethics Committee.

### Experimental paradigm

**Fig. 1:**
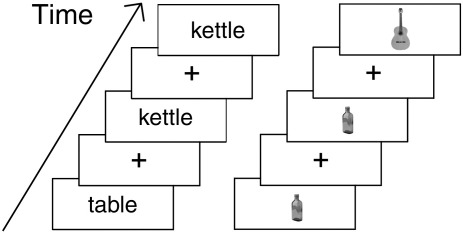
The 1-back paradigm used to functionally localise word and object-sensitive regions. Note that this image is not to scale. Words were presented in 32 pt Helvetica font and subtended a visual angle of 4°. Pictures were 250 × 250 pixels and subtended a visual angle of 4°.

In order to investigate intra- and inter-subject consistency for reading and object sensitivity in ventral and lateral OTC respectively, a one-back task was used with four categories of visual stimuli: written words, pictures of common objects, scrambled pictures of the same objects, and consonant letter strings (Fig. 1). Subjects were instructed to press a button if the stimulus was identical to the preceding stimulus and 12.5% of the stimuli were targets. A block design was used to maximize statistical sensitivity. Each block consisted of 16 trials from a single category presented one every second. A trial began with a 650 ms fixation cross, followed by the stimulus for 350 ms. In between blocks, subjects viewed a fixation cross for 16 s. The stimuli were divided equally into two lists, with the order counter-balanced across subjects such that 50% of subjects saw the first list of stimuli during run 1 and the remaining 50% during run 2. In total there were 192 stimuli per category including targets. Using a one-back task has the advantage that stimulus category can be varied without changing the task, maintaining a constant cognitive set — the specific stimuli are almost incidental to the task. In addition, it is commonly used for functional localisation (Baker et al., 2007; Downing et al., 2007; Gazzaley et al., 2005; Kanwisher et al., 1999; Peelen and Downing, 2005).

Word stimuli (*n* = 168) were obtained from the MRC psycholinguistic database (Coltheart, 1981) and consisted of 4 or 5 letter words with regular spellings (e.g. “hope”). All words had familiarity ratings between 300 and 500 (Coltheart, 1981), were either one or two syllables, and had a British English written word frequency value of 40 or less (Baayen et al., 1993). The stimuli in the two runs were fully matched for frequency, familiarity, imageability, number of letters, and number of syllables. Object stimuli consisted of black and white pictures (200 × 250 pixels) of easily recognizable objects such as a boat, tent, nail, etc. The scrambled objects were generated by dividing the pictures into 10 × 10 pixel squares and permuting their placement within the image. None of the resulting images were recognizable after scrambling. Finally, consonant letter strings were unpronounceable strings randomly generated to exactly match the length of the word stimuli.

### Functional imaging

Whole-brain imaging was performed on a Siemens 1.5 T MR scanner at the Birkbeck-UCL Neuroimaging (BUCNI) Centre in London. The functional data were acquired with a gradient-echo EPI sequence (TR = 3000 ms; TE = 50 ms; FOV = 192 × 192; matrix = 64 × 64) giving a notional resolution of 3 × 3 × 3 mm. Each run consisted of 164 volumes and as a result, the two runs together took 16.4 min. In addition, a high-resolution anatomical scan was acquired (T1-weighted FLASH, TR = 12 ms; TE = 5.6 ms; 1 mm^3^ resolution) for anatomically localising activations in individuals.

Data processing was carried out using FSL 4.0 (www.fmrib.ox.ac.uk/fsl). To allow for T1 equilibrium, the initial two images of each run were discarded. The data were then realigned to remove small head movements (Jenkinson et al., 2002), smoothed with a Gaussian kernel of FWHM 6 mm, and pre-whitened to remove temporal auto-correlation (Woolrich et al., 2001). The resulting images were entered into a general linear model with four conditions of interest corresponding to the four categories of visual stimuli. Blocks were convolved with a double gamma “canonical” hemodynamic response function (Glover, 1999) to generate the main regressors. In addition, the estimated motion parameters were entered as covariates of no interest to reduce structured noise due to minor head motion. Linear contrasts of [words > fixation] and [objects > scrambled objects] identified reading- and object-sensitive areas, respectively. First level results were registered to the MNI-152 template using a 12-DOF affine transformation (Jenkinson and Smith, 2001) and all subsequent analyses were conducted in the MNI standard space. A second level fixed-effects model combined the two first level runs into a single, subject-specific analysis which was then entered into a third level, mixed effects analysis to draw inferences at the population level (Beckmann et al., 2003; Woolrich et al., 2004).

Note that consonant strings were originally intended to serve as a baseline condition for words analogous to scrambled pictures for objects. Although the contrast [words > consonants] produced activation in vOTC at the random effects level similar to previous studies (Cohen et al., 2002; Devlin et al., 2006), the activation was not reliable for individuals (see also Baker et al., 2007; Cohen et al., 2002; Vigneau et al., 2005) and therefore [words > rest] was used to identify reading-sensitive areas instead.

### Regions-of-interest

In order to restrict the analyses to the ventral and lateral OTC, two anatomical masks were drawn in standard space. The ventral OTC mask encompassed the posterior portion of the left fusiform gyrus, occipito-temporal sulcus (OTS), and medial parts of the inferior temporal gyrus (ITG) — areas consistently activated by visual word recognition tasks (Fiez et al., 1999; Fiez and Petersen, 1998; Herbster et al., 1997; Price et al., 1996; Price et al., 1994; Rumsey et al., 1997; Shaywitz et al., 2004). The standard space coordinates were: *X* = − 30 to − 54, *Y* = − 45 to − 70 and *Z* = − 30 to − 4. This region is sometimes referred to as the “visual word form area” (Dehaene et al., 2005; McCandliss et al., 2003), although the term is misleading as it suggests a functional specificity which is not present (Price and Devlin, 2003, 2004). The lateral OTC mask encompassed lateral posterior fusiform gyrus, posterior OTS and lateral parts of posterior ITG — areas consistently activated by visual objects and collectively known as the “lateral occipital complex” (Grill-Spector et al., 1999; Malach et al., 1995). The standard space coordinates were *X* = − 33 to − 56, *Y* = − 67 to − 89 and *Z* = − 20 to + 4. Within each mask, only voxels with at least a 20% chance of being grey matter were included based on an automatic tissue segmentation algorithm (Zhang et al., 2001).

## Results

### Behaviour: 1-back performance

Behavioural data from six subjects were lost due to a problem recording button press responses while in the scanner. The data from the remaining subjects (*n* = 39) were analysed using signal detection theory as hits and false alarms. The mean hit rate was 0.791 and the false alarm rate was 0.011, indicating that participants performed the task adequately (see Table 1). In addition, d-prime (d') scores were calculated to measure sensitivity for detecting repeated items (Table 1). These were then entered into 4 × 2 repeated measures ANOVA examining the effects of Category (words, consonant strings, objects, scrambled objects) and Run (first, second). A main effect of Category (*F*(3,114) = 77.9, *p* < 0.0001) indicated that detecting repetitions of scrambled objects was most difficult, but there was no difference between words or objects (*t*(38) = 0.05, *p* = 0.961). Importantly, neither the main effect of Run (*F*(1,38) = 0.494, *p* = 0.486) nor the Category × Run interaction (*F*(3,114) = 1.665, *p* = 0.179) was significant, indicating that participants' performance did not significantly change from the first to the second run. The same pattern was present in the reaction times to correct detections (i.e. “hits”). Again, there was a main effect of Category (*F*(3,114) = 5.4, *p* = 0.002) but no main effect of Run (*F*(1,38) = 0.09, *p* = 0.765) and no Category × Run interaction (*F*(3,114) = 1.169, *p* = 0.325). In other words, there was no behavioural evidence for task learning that might confound the activation patterns across runs.

## Imaging results

### Group effects

**Fig. 2:**
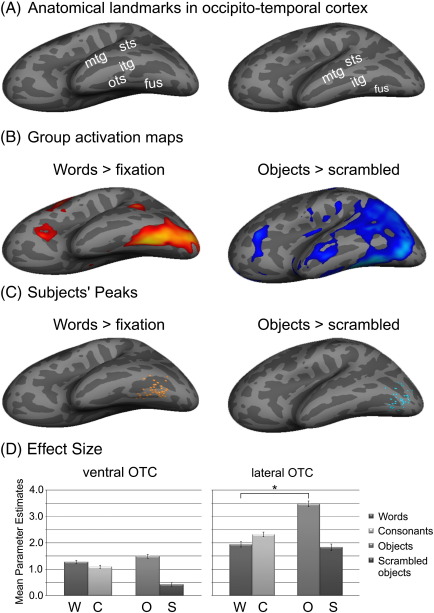
Results of functionally localising word- and object-sensitive areas of occipito-temporal cortex. (A) An inflated left hemisphere of a single brain illustrating the main anatomical landmarks in the OTC. Sulci are shown in dark gray and gyri in light gray. (B) Group activation for [words > fixation] and [objects > scrambled] projected on to the inflated left hemisphere of the Freesurfer “fsaverage” brain. (C) The spatial distribution of individual subject peaks for [words > fixation] in ventral OTC (orange dots) and for [objects > scrambled] in lateral OTC (blue dots). Note that the sharp demarcations between gyri and sulci do not accurately reflect the anatomical variability present in the group. Instead the figure illustrates the spatial distribution of peaks relative to a single brain. (D) Effect sizes for words, consonant strings, objects and scrambled objects relative to fixation in ventral and lateral OTC. Error bars represent standard error of the mean. ⁎ indicates a significant difference at *p* < 0.001. Abbrevs: mtg = middle temporal gyrus, sts = superior temporal sulcus, itg = inferior temporal gyrus, ots = occipito-temporal sulcus, fus = fusiform gyrus; W = words, C = consonant strings, O = objects, and S = scrambled objects.

Consistent with previous research, the peak activation in ventral OTC for words relative to fixation was located in the occipito-temporal sulcus (− 42, − 50, − 20; *Z* = 7.7), extending both medially onto the convexity of the posterior fusiform gyrus and laterally onto the inferior temporal gyrus. To visualize this activation, the group results were projected onto an inflated surface of an “average” brain (i.e. Freesurfer's fsaverage subject) to illustrate that activation was not limited to the ventral surface but also present inside the occipito-temporal sulcus (Fig. 2B). As reported previously (Bookheimer et al., 1995; Moore and Price, 1999; Price et al., 2006; Wright et al., 2008), objects relative to scrambled objects also activated this same region (− 40, − 58, − 20; *Z* = 7.9; Fig. 2D) and although activation for objects was numerically larger than for words, there was no significant difference between them. Within the lateral OTC, objects produced strong activation in LOC (− 41, − 78, − 9; *Z* = 7.5), although once again, there was a comparable activation for words (− 37, − 84, − 11; *Z* = 6.9; Fig. 2B). Here, objects did lead to significantly greater activation than words (*Z* = 5.9; Fig. 2D), but this was part of a much larger cluster encompassing almost the entire occipital lobe and extending ventrally through large parts of the inferior temporal lobe bilaterally (c.f. Moore and Price, 1999). In other words, the group results demonstrate that the task and stimuli were appropriately able to identify ventral and lateral OTC areas and confirm previous studies that demonstrate greater activation for objects than words in OTC regions (Bookheimer et al., 1995; Moore and Price, 1999; Price et al., 2006; Wright et al., 2008).

### Inter-subject variability

To assess how closely activation from individuals matched the group results, their peak responses for words and objects were compared to the group results. For words, all 45 participants showed a peak response within ventral OTC with a *Z*-score of at least 3.5, although the specific location varied considerably (Table 2). The left panel of Fig. 2C illustrates the spatial distribution of peaks within ventral OTC. Individual subject peaks are shown as orange dots. Each peak has been projected onto a single brain that has been inflated to show not only the crests of the gyri (light grey) but also the depths of the sulci (dark grey) using Freesurfer (http://surfer.nmr.mgh.harvard.edu/). Note that the sharp demarcations between gyri and sulci do not accurately reflect the anatomical variability present in the group; instead the figure illustrates the spatial distribution of peaks relative to a single “average” brain. Consequently, the specific anatomical location of each peak was assessed relative to that individual's structural scan in standard space. The greatest consistency is in the medial-lateral direction, with the majority of peaks (*n* = 20) falling within the occipito-temporal sulcus. Another 18 were located on the crest of the posterior fusiform gyrus and 7 were in on the crest of the inferior temporal gyrus. In contrast, the largest variation was in the rostro-caudal direction while the variation in *z*-axis is mostly due to the depth of the OTS. On average, the Euclidean distance from an individual subject's peak to the group peak was 15 mm (± 5 mm).

There was slightly less variability in the peak coordinates for objects within lateral OTC. Once again, all 45 participants showed a clear peak in the ROI with *Z*-scores of 2.7 or higher and these are illustrated in the right panel of Fig. 2C. The majority of peaks for objects lay in lateral occipital cortex (*n* = 26) and the remaining ones were located in posterior fusiform cortex (*n* = 19). Unlike the reading peaks, these were spread more evenly around group peak and on average, the Euclidean distance from an individual subject's peak to the group peak was 9 mm (± 3 mm).

### Intra-subject variability

The most critical analyses for evaluating the consistency assumption underlying functional localisers concerned within-subject consistency. This was calculated in three ways. Because studies often define functional ROIs using a sphere with a fixed radius centred on the peak voxel (Blankenburg et al., 2006; Jiang et al., 2007; Miller and D'Esposito, 2005; Pulvermüller et al., 2006), the first measure examined the spatial reliability of the peak voxel since this determines the fROI. The coordinates of peak voxels were extracted for each participant from both runs and the distance between peaks was calculated using the standard Euclidean distance measurement. On average, peaks for words were separated by 7.4 mm while peaks for objects were 8.3 mm apart. It is worth noting that at the resolution of the acquired data (3 × 3 × 3 mm), these peaks would be 2–3 voxels apart in space, although this figure varied considerably across participants. A number of subjects showed peaks within 1 voxel of each other (words: *n* = 17; objects: *n* = 11) however many subjects had peaks more than 4 voxels (> 12 mm) apart (words: *n* = 12; objects: *n* = 12). The coordinates of the peak depend on many factors, however, and only one is the size of the underlying neurophysiological response. Therefore, peak locations are highly susceptible to random fluctuations (Aguirre et al., 1998; Handwerker et al., 2004; Kruger and Glover, 2001). Consequently, the second analysis focused on the set of voxels within the ROI that were activated by both runs.

**Fig. 3:**
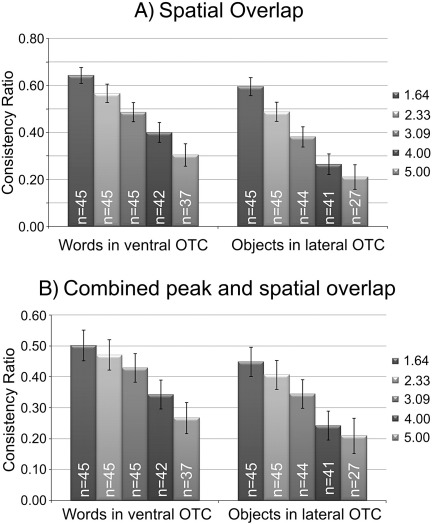
Consistency of fROI activation when different statistical thresholds were used to define “active” voxels. (A) These bar plots show consistency scores based on spatial overlap between the two localiser runs. Activation thresholds ranged from lenient (*Z* > 1.64, *p* < 0.05 uncorrected) to conservative (*Z* > 5.0, *p* < 10^− 6^). At the lowest thresholds, all participants had active voxels in the ROI but as the threshold increased, some subjects needed to be excluded from the analyses due to lack of activation at that threshold. The numbers in white refer to the number of subjects who were included in the analysis at each level (out of 45). (B) In these plots, consistency was evaluated on the set of contiguously activated voxels within 9 mm of the peak response. Error bars represent standard error of the means.

**Fig. 4:**
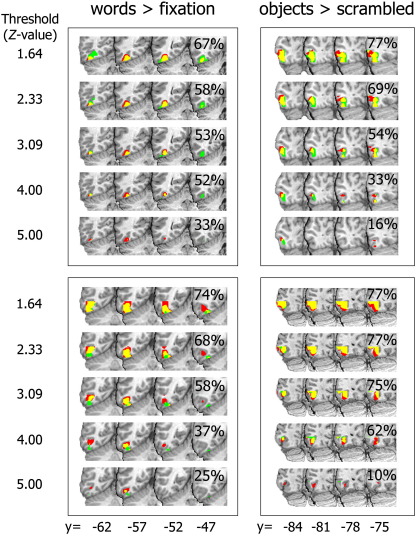
An illustration of how consistency interacts with statistical thresholding in two representative participants. The left column shows activation for [words > fixation] in the left ventral occipito-temporal region for two runs at multiple thresholds while the right column shows the same for [objects > scrambled] in the left lateral occipital complex. Voxels that were only activated in the first and second runs are coloured green and red, respectively, while voxels that were active in both runs are coloured yellow. Note that increasing the threshold decreases the overlap.

The most common method for defining an fROI is based on the volume of activated voxels within a particular region (Grill-Spector et al., 2004; Jiang et al., 2007; Kanwisher et al., 1999; Spiridon et al., 2006; Szycik et al., 2008; Yovel et al., 2008). Consequently, the second measure assessed consistency in terms of the volume of commonly activated cortex between runs in both ventral and lateral OTC. This was computed as the ratio (*R*_ij_) of commonly activated voxels to the total number of activated voxels in two runs, i and j:

(1) $$R_{\text{ij}}=2\times V_{\text{ij}}/\left(V_{\text{i}}+V_{\text{j}}\right)$$

where *V*_ij_ is the number of voxels within the ROI which were active in both runs i and j; and *V*_i_ and *V*_j_ are the number of voxels within the ROI that were active in runs i and j, respectively. A value of 1.0 indicates identical sets of voxels while 0.0 represents completely disjoint sets. This definition, however, treats voxels as “active” or not based on an essentially arbitrary threshold. To avoid conditioning the results by an arbitrary choice, five thresholds were used spanning a typical range: i) *Z* > 1.64 (*p* < 0.05 uncorrected), ii) *Z* > 2.3 (*p* < 0.01 uncorrected) iii) *Z* > 3.09 (*p* < 0.001 uncorrected), and iv) *Z* > 4.0, (roughly *p* < 10^− 4^, which is fairly conservative) and v) *Z* > 5.0 (roughly *p* < 10^− 6^, which would conservatively correct for multiple comparisons across the whole brain with a family-wise α < 0.05). Mean (± SEM) consistency ratios were similar in both ventral and lateral OTC regions with the highest values (0.64 ± 0.03 and 0.60 ± 0.04) for the lowest statistical threshold (Fig. 3A). Raising the statistical threshold *decreased* the amount of overlap between runs, and this is illustrated in Fig. 4. In this figure, data from two representative subjects show how the increasingly conservative statistical threshold influences the overlap (yellow) between runs (shown in red and green). At lenient thresholds, there is widespread activation within both the ventral and lateral occipito-temporal ROIs, leading to considerable overlap (the consistency score is shown in the upper right corner of the panel). At higher thresholds, however, two things typically happened. First, the number of active voxels in one or both runs decreased dramatically, reducing the overlap between runs. Second, the active clusters from the two runs tended to separate spatially, leaving only a small region of common activation. At the two most conservative statistical thresholds (*Z* > 4.0 and *Z* > 5.0) the mean consistency scores were 0.30 ± 0.05 and 0.21 ± 0.05, respectively. In addition, higher thresholds meant fewer subjects with significantly activated voxels. For instance, at the most conservative threshold it was impossible to identify an fROI for words or objects in 8 and 18 (out of 45) participants, respectively. In sum, overlap scores were surprisingly low with more conservative statistical thresholds yielding even less overlap and fewer subjects in which an fROI could be defined.

Finally, it is possible to combine peak and volume measures to define an fROI as the set of active voxels that are contiguous with the peak activation (Downing et al., 2006). This approach will help to reduce variability between runs as long as the two peaks fall within overlapping clusters. To assess the consistency of this method, we defined fROIs as the set of contiguous active voxels (using the same set of five thresholds as above) that included the peak voxel and were within a 9 mm radius of the peak voxel following Downing et al.(2006). The results are shown in Fig. 3B. Again, the highest consistency values were for the lowest statistical threshold. For the contrast [words > fixation] in ventral OTC, the mean consistency ratio (*R*_ij_) was 0.50 (SEM = 0.05) and for the contrast [objects > scrambled], the mean *R*_ij_ was 0.45 (SEM = 0.05). Increasing the threshold to *Z* > 5 reduced the overlap to 0.27 ± 0.05 for words and 0.21 ± 0.06 for objects and precluded identifying an fROI in 8 and 18 of the participants.

## Discussion

The aim of this study was to evaluate the consistency associated with functionally localising reading- and object-sensitive areas of left occipito-temporal cortex. At the group level, the current results closely match previous reports with peak activations located in the posterior occipito-temporal sulcus for written words (Ben-Shachar et al., 2007; Cohen et al., 2000; Devlin et al., 2006; Kronbichler et al., 2004; Price and Mechelli, 2005; Shaywitz et al., 2004) and in the lateral occipital region for visual objects (Grill-Spector et al., 1999; Malach et al., 1995). In other words, the ability to localise these regions at the group level is highly consistent across studies. At the individual level, however, localization was considerably less consistent, with peaks varying in location by as much as 20 mm in any direction. This finding replicates previous studies and demonstrates the importance of using functional data to localise a specific region-of-interest when characterizing its response properties (Kanwisher et al., 1997; Saxe et al., 2006a; Wright et al., 2008). But in order for functional localisation to be meaningful, it must be robust and consistent *within* subjects. The current findings suggest that this consistency was surprisingly poor, regardless of the specific method used to evaluate consistency:

1. *Peak voxels*. Roughly 33% of our participants had peaks at essentially the same location in both localiser runs whereas another 27% had peaks that were at least 12 mm apart. The remainder fell within those two extremes. In other words, for one quarter of the subjects tested here, an fROI based on the peak voxel response may not even overlap with the activation seen in the main experimental task.
2. *Spatial overlap*. The most commonly used method for defining an fROI is to select the voxels within a region activated by a given contrast. Clearly this depends critically on the definition of “active voxels” and this varies from study to study. Over a wide range of activation thresholds (*p* < 0.05 to *p* < 10^− 6^), consistency scores were surprisingly low, ranging from 64% to 21%, respectively. The most lenient definition of “active” voxels produced the greatest consistency across runs, but even so, roughly one third of the data from the fROI are coming from noisy or unreliable voxels. Equally problematic is the fact that lenient statistical thresholds lead to only minimal category-selectivity in the ROI (Fox et al., 2008; Golarai et al., 2007). Conservative statistical thresholds (*p* < 10^− 3^ to 10^− 6^) are more common but yield very low consistency values, with less than half of the voxels present in both runs. As a result, the majority of the data being investigated comes from unreliable voxels.
3. *Peak plus spatial extent*. In theory, combining the first two methods has the advantage that small displacements of the peak voxel do not necessarily change the fROI, assuming they fall within a common cluster of active voxels. In practice, the results were qualitatively and quantitatively similar to the previous method because of the small numbers of active voxels common to both runs.

One potential explanation for the low levels of overlap between runs is that participants may acclimate to the task and therefore show less activation in their second run. There was, however, no evidence of task learning in the behavioural data. This was also true for the imaging data where we analysed the number of active voxels per contrast with Run (first, second) and Threshold (1.64, 2.33, 3.09, 4.0, 5.0) as independent factors. Predictably there was a main effect of Threshold on the number of active voxels for both contrasts (words: *F*(4, 179) = 229.3, *p* < 0.001; objects: *F*(4, 179) = 192.9, *p* < 0.001). but there was no main effect of Run (words: *F*(1,44) < 0.1, *p* = 0.936; objects *F*(1,44) = 1.2, *p* = 0.287) and no Run × Threshold interaction (words: *F*(4,176) = 0.3, *p* = 0.896; objects: *F*(4,176) = 0.8, *p* = 0.520). In other words, task learning did not appear to significantly contribute to the relatively low consistency between runs.

The single largest source of variability appeared to be spatial shifts in activation (see Fig. 4), which help to explain the surprising finding that overlap decreased with more conservative statistical thresholding (see also Kung et al., 2007). Initially, we assumed that higher statistical thresholds would converge on the most selective category-sensitive voxels which we expected would be stable across runs. In practice, however, the highest thresholds showed the lowest consistency scores resulting in a trade-off between category-selectiveness and consistency. One potential limitation of our study is that we only tested two types of category-selectivity and only in two anatomical areas, so it is possible that our results may not generalize to other areas. On the other hand, despite being sensitive to different categories of stimuli, both regions showed essentially the same pattern and this pattern matched those of Kung et al. (2007) who found at best 50% consistency in face-sensitive areas.

This variability for category-sensitive visual areas stands in contrast to the consistency seen for retinotopically-defined visual areas, which appear remarkably stable within individuals (M. I. Sereno, personal communication). One striking difference between retinotopic vs. category-sensitive localisers is the amount of data typically collected. Studies of retinotopy often collect an order of magnitude more data (see Table 2). For instance, it is not uncommon to functionally localise category-sensitive regions based on one or more scans that take a total of 20 min or less. In contrast, retinotopy is typically defined using six to twelve scans that together take an hour or more, so perhaps it is not surprising that the results are more consistent. On other hand, it is possible that the observed variability in visual association areas accurately reflects functional-anatomic variability in these regions due to neuronal firing patterns becoming increasingly distant from the stimulus they are intended to represent. Either way, this issue has important implication for the design of functional localiser scans because it suggests that additional data will be necessary to maintain sensitivity and avoid experimental bias or it places absolute limits on the reliability of functional localisers. Clearly further work is necessary to resolve this question (Table 3).

In short, these findings call into question the reliability of functional localisers to identify a meaningful and consistent set of category-sensitive voxels. One of the prime motivations for using functional localisers is to maximize sensitivity by explicitly accounting for inter-subject variability in the location of a functionally defined region (Saxe et al., 2006a). Our results suggest, however, that it is equally important to explicitly consider intra-subject variability when defining an fROI to avoid analyzing data from voxels that are not consistently present in the fROI.

### Implications for TMS

Finally, our results also have important implications for TMS studies. There are several options when choosing a method for targeting stimulation, including using fMRI-based neuro-navigation, using standard space coordinates from published imaging studies, or using heuristic methods such as the 10–20 system. Recent empirical studies have shown that although all three methods work, the latter two are sub-optimal, requiring higher stimulation intensities and/or larger numbers of subjects (Sack et al., 2009; Sparing et al., 2008). This is almost certainly due to the considerable inter-subject variability in the location of peak responses (current results; Kanwisher et al., 1997; Wright et al., 2008) rendering stimulation based on group coordinates less efficient. This problem is further compounded by a heuristic approach to targeting because of the inherent variability between the measurement system (e.g. the 10–20 system) and the underlying anatomy (Steinmetz et al., 1989). Clearly, an optimal targeting method will take into account inter-subject variability either through neuro-navigated TMS (Andoh et al., 2006; Duncan et al., in press) or by localising the stimulation directly with TMS (Devlin et al., 2003; Gough et al., 2005). Our current results suggest that even neuro-navigated TMS is not an entirely reliable method due to the localisation variability within a single subject, particularly when coupled with spatial distortions in EPI images due to magnetic field inhomogeneities and draining veins (Devlin et al., 2003; Duong et al., 2001; Jezzard and Balaban, 1995; Terao et al., 1998; Turner, 2002). Instead, a TMS-based functional localiser probably represents the optimal method for targeting stimulation as it avoids all of these sources of error and provides a direct measure of the effect of stimulation across a range of target sites.

### Conclusions

The current findings demonstrate surprisingly low intra-subject consistency when functionally localising word- and object-sensitive regions of occipito-temporal cortex but also highlight considerations for designing and reporting studies using functional localisers. The most obvious is simply optimising data collection wherever possible. Collecting larger quantities of localiser data, reducing sources of variability (Friston et al., 2006), and optimising both stimuli and tasks (Fox et al., 2008) will all improve the consistency of the results. It is worth noting that the use of factorial designs, rather than separate localiser scans, will also improve consistency by using the same data to define the fROI as is used to interrogate the response profile of the region (Friston et al., 2006). Finally, clearer reporting of the exact methods used to functionally localise a region would also assist readers in evaluating the robustness of the findings. At a minimum, these could include clear information about the amount of data collected as well as the details of how the fROI was defined. For instance, in many cases it is unclear what anatomical criteria (if any) are used to limit the extent of the fROI to the region under investigation. Functional localisers currently play an important role in cognitive neuroscience, and no doubt will become even more important in the future. Consequently, it will be increasingly important to optimise the practice to provide consistent localisation within individuals in order to maximize sensitivity and avoid potential sources of bias.

## Figures and Tables

**Table 1:** Behavioural data from the 1-back task. The top section presents overall performance in terms of hit and false alarm rates. The second section presents sensitivity scores (d'-values) for detecting item repetitions while the third presents the reaction times for correctly detecting repeated items.

	Words	Consonants	Objects	Scrambled
Hit rate	0.866	0.842	0.854	0.602
False alarms	0.006	0.006	0.002	0.030
D-Prime scores (± SEM)				
Run 1	4.03 (0.15)	3.96 (0.13)	4.05 (0.13)	2.43 (0.14)
Run 2	4.16 (0.15)	3.83 (0.14)	4.15 (0.13)	2.64 (0.14)
Median RTs (SEM)				
Run 1	582 (14.1)	560 (13.0)	568 (13.2)	599 (15.6)
Run 2	578 (11.9)	562 (12.7)	585 (11.7)	600 (13.1)

**Table 2:** Summary of inter-subject variability in peaks coordinates for words and objects. Coordinates are in the MNI152 space and the *Z*-score is for the peak voxel.

	*X*	*Y*	*Z*	*Z*-score	Distance to group peak (mm)
Words in ventral OTC					
Range	− 52…− 30	− 70…− 46	− 25… − 5	3.5…12.7	5.7…23.9
Mean	− 42	− 62	− 16	8.8	15.2
S.D.	5	7	5	2.5	5.2
Objects in lateral OTC					
Range	− 55…− 34	− 87…− 68	− 19…3	2.7…13.9	4.1…18.5
Mean	− 43	− 77	− 8	7.1	9.2
S.D.	5	6	6	2.4	3.5

**Table 3:** Summary of functional localiser scans from selected studies. Note that this is not intended to be an exhaustive list of studies employing functional localisers but a representative sample.

Study	Localising	Task	Vols per condition	Runs
Early visual areas				
Sereno et al. (1995)	Retinotopy	Passive viewing	128	8
Tootell et al. (1997)	Retinotopy	Passive viewing	128	6–12
Larsson and Heeger (2006)	Retinotopy	Passive viewing	168	10
Higher order visual areas and non-visual areas				
O'Craven and Kanwisher (2000)	Faces	Naming	60	2
Haxby et al. (2001)	Faces, objects, etc.	1-back	10	12
Yovel et al. (2008)	Faces, objects, etc.	1-back	64	1
Levy et al. (2001)	Faces, objects, etc.	1-back	21	1
von Kriegstein et al. (2008)	Faces, objects	Passive viewing	25	2
Szycik et al. (2008)	Speech	Semantic decision	90	1
Pulvermüller et al. (2006)	Motor	Movement	40	1
Blankenburg et al. (2006)	Somatotopy	Cutaneous stimulation	38	1
Saxe and Kanwisher (2003)	Theory of mind	ToM task	60	3
Jiang et al. (2007)	Faces, cars	Passive viewing	30	2
Baker et al. (2007)	Words	Passive viewing/1-back	40	4

## Footnotes

1 This value was based on Kung et al.'s (2007) Fig. 5a and uses a formula sensitive to differences in the number of active voxels between runs (as is used in our own analyses). The overlap value increases when this difference in voxel numbers ignored but even so, remains less than 50%.
